# Spatio-temporal patterns and movement analysis of pigs from smallholder farms and implications for African swine fever spread, Limpopo province, South Africa

**DOI:** 10.4102/ojvr.v82i1.795

**Published:** 2015-11-27

**Authors:** Folorunso O. Fasina, Japhta M. Mokoele, B. Tom Spencer, Leo A.M.L. van Leengoed, Yvette Bevis, Ingrid Booysen

**Affiliations:** 1 Department of Production Animal Studies, University of Pretoria, South Africa; 2 Limpopo Department of Agriculture, Groblersdal, South Africa; 3 Department of Farm Animal Medicine, Utrecht University, the Netherlands; 4 Centre for Geoinformation Science, Department of Geography, Geoinformatics and Meteorology, University of Pretoria, South Africa

## Abstract

Infectious and zoonotic disease outbreaks have been linked to increasing volumes of legal and illegal trade. Spatio-temporal and trade network analyses have been used to evaluate the risks associated with these challenges elsewhere, but few details are available for the pig sector in South Africa. Regarding pig diseases, Limpopo province is important as the greater part of the province falls within the African swine fever control area. Emerging small-scale pig farmers in Limpopo perceived pig production as an important means of improving their livelihood and an alternative investment. They engage in trading and marketing their products with a potential risk to animal health, because the preferred markets often facilitate potential long-distance spread and disease dispersal over broad geographic areas. In this study, we explored the interconnectedness of smallholder pig farmers in Limpopo, determined the weaknesses and critical control points, and projected interventions that policy makers can implement to reduce the risks to pig health. The geo-coordinates of surveyed farms were used to draw maps, links and networks. Predictive risks to pigs were determined through the analyses of trade networks, and the relationship to previous outbreaks of African swine fever was postulated. Auction points were identified as high-risk areas for the spread of animal diseases. Veterinary authorities should prioritise focused surveillance and diagnostic efforts in Limpopo. Early disease detection and prompt eradication should be targeted and messages promoting enhanced biosecurity to smallholder farmers are advocated. The system may also benefit from the restructuring of marketing and auction networks. Since geographic factors and networks can rapidly facilitate pig disease dispersal over large areas, a multi-disciplinary approach to understanding the complexities that exist around the animal disease epidemiology becomes mandatory.

## Introduction

Infectious and zoonotic diseases outbreaks have intensified in past decades through more intense interconnectedness, rapid transport, the opening-up of borders and increasing volumes of legal and illegal trades (Jones *et al*. 2013; Perry, Grace & Sones 2013). Spatio-temporal analyses have been used in veterinary medicine and other fields in recent times (Jiang, Ediger & Bader 2009; Paul & Dasgupta 2012; Rivas *et al*. 2012). Specifically, geographic factors such as roads, water bodies, distances from other outbreaks and markets, among other factors, have been found to play important roles in disease transmission (Food and Agriculture Organization of the United Nations [FAO] 2013; Jori *et al*. 2009; Korennoy *et al*. 2014; Pastrana *et al*. 2014; Rivas *et al*. 2010Rivas *et al*. 2012; Sánchez-Vizcaíno, Mur & Mártinez-López 2012). In addition, trade practices have played major roles in the spread of infectious diseases among livestock (Fournié *et al*. 2013; McCarron *et al*. 2015).

Limpopo province is very important in the epidemiology of some transboundary animal diseases in South Africa. In particular, the province has been designated as a control zone for African swine fever together with certain areas of the North West and Mpumalanga provinces (Department of Agriculture, Fisheries and Forestry [DAFF] n.d.; Penrith 2013). Furthermore, the province provides an active domestic animal–wildlife–human interface, making it an ideal location for One Health studies involving zoonoses or disease interactions between wildlife and domestic animals.

Pig production systems and the particular contributions of the emerging small-scale pig farms (ESSPF) in the Limpopo have been described recently (Mokoele *et al*. 2014). However, the presence of a deeprooted dual market structure remains a major challenge facing small-scale pig producers in the province (Antwi & Seahlodi 2011). While the commercial pig farmers use the formal markets (standard abattoirs, processing plants and supermarkets), the ESSPF farmers can access only the informal markets (local auctions, backyard slaughter, pension sale points and local abattoirs/slaughter slabs).

The most popular auction point and abattoir used by the ESSPFs from Limpopo are Belfast (Mpumalanga) and Bronkhorstspruit (Gauteng) respectively. These locations, which are outside the province, present the ESSPFs with better marketing opportunities and higher incomes for their products. While the commercial interests of the ESSPFs are being secured through these more affluent markets, breach of biosecurity remains evident and the risk of introducing infectious pathogens to non-endemic areas remains high, with imminent threats to the pig industry nationally. Martínez-López, Pérez and Sánchez-Vizcaíno (2009) and Lindström *et al*. (2009) have previously identified such risks elsewhere.

ASF is a severe, highly transmissible viral infection of domestic pigs that manifests itself as a haemorrhagic fever, which can cause mortality of up to 100% in domestic pigs with consequent devastating effects on the livelihoods of farmers who depend on pig production (Bastos, Fasina & King 2014; de Glanville *et al*. 2014; Fasina *et al*. 2012a; Penrith 2013). To date, three types of epidemiological cycle have been described for ASF in southern Africa (Penrith, Thomson & Bastos 2004).

### Historical perspective on the disease in South Africa with emphasis on Limpopo

The first documented outbreak of ASF in South Africa was recorded in 1928 and this was related to contact between wild pigs and domestic pigs (Penrith 2013; Penrith & Vosloo 2009). Between 1933 and 1939, historical outbreaks of swine fever were documented (De Kock, Robinson & Keppel 1940); cases may have been classical or African swine fever, as no clear distinction was established between the two diseases in earlier years (Penrith 2013). Since 1939, South Africa has experienced only sporadic outbreaks that have been limited to the ASF-controlled zones of Limpopo and the Kruger National Park (Boshoff *et al*. 2007). Although parts of the North West province are designated within the control zones, ASF had not been reported there (DAFF 2014, Table 1). Recent events have, however, proved that the virus, although contained, has the capacity to spread rapidly from the controlled areas to new locations within South Africa (Table 1).

**TABLE 1:** Reported outbreaks of ASF, 1993–2012 in South Africa

Timeline	Years	Province	Outbreaks		Cases		Dead/euthanased		References
						*n*	%	*n*		%	*n*	%	
Recent	1993–2012	Limpopo	54	76.1	1040	79.4	1258	64.1	Evans (2012), Spencer(2012), Penrith and Vosloo (2009), Spencer (2012) and DAFF (2014)
		Mpumalanga	9	12.7	133	10.2	585	29.8	
		Gauteng	6	8.4	132	10.1	116	5.9	
		Kruger National Park	2	2.8	4	0.3	4	0.2	
		National	71	-	1309	-	1963	-	

Note: Please see the full reference list of the article, Fasina, F.O., Mokoele, J.M., Spencer, B.T., Van Leengoed, L.A.M.L., Bevis, Y. & Booysen, I., 2015, ‘Spatio-temporal patterns and movement analysis of pigs from smallholder farms and implications for African swine fever spread, Limpopo province, South Africa’, *Onderstepoort Journal of Veterinary Research* 82(1), Art. #795, 11 pages. http:// dx.doi.org/10.4102/ojvr.v82i1.795, for more information.

In South Africa between 1993 and 2012, 1309 cases of ASF were documented between 1993 and 2012 from 71 outbreaks in South Africa (Table 1; DAFF 2014). Limpopo has accounted for 76.1% of all outbreaks to date and the majority of outbreaks from other provinces have links with Limpopo (Table 1).

In September–October 2011, Mpumalanga and Gauteng experienced the first outbreak of ASF to occur outside the control area in recent times. Briefly, some pigs died in a small piggery within the ASF control area in Lephalale (Ellisras), Limpopo with the subsequent sale of surviving pigs at an auction in Sundra, Mpumalanga, and their transportation to Rietpoort abattoir in Gauteng. By January 2012, a total of 172 farms including some 10 374 pigs have had primary or secondary contacts with the index farm and only the prompt and co-ordinated intervention of the meat inspectors, private and government veterinarians, the industry and the laboratory (Transboundary Animal Diseases Programme (TADP)) curtailed the outbreaks and eradicated the infections (Evans 2012; Spencer 2012; Spencer & Penrith 2014).

In this study, we used the spatio-temporal data of locations, possible interactions and marketing structures practised by the emerging small-scale farming communities in Limpopo to draw a network of ESSPF, conduct some spatio-temporal analyses and integrate the outcomes with historical and recent reports of ASF outbreaks in South Africa to map the probable risk, spread and consequences of ASF. In addition, because Limpopo has played a significant role in the outbreaks and dissemination of ASF in South Africa and the province has been declared an ASF-endemic location, we used ASF as a model for a rapidly spreading transboundary animal disease amongst pig populations.

## Materials and methods

### Data collection

As part of the questionnaire survey conducted for a study on the production systems and dynamics of the ESSPF in Limpopo (Mokoele *et al*. 2014), the geo-coordinates of all the surveyed ESSPF were obtained using the Garmin Nuvi^®^ or the Nokia Lumia 635^®^ and entered into a Microsoft Excel^®^ spreadsheet. Briefly, Limpopo consists of five districts, namely Sekhukhune, Capricorn, Waterberg, Vhembe and Mopani. A purposive sampling method was targeted at all the available ESSPF farmers (*n* = 185) in these districts.

An original list of 85 small-scale pig farmers was produced by the Limpopo Department of Agriculture but an additional 100 farms that fall within this category but were not listed were recruited into the survey because the preliminary evidence from the field suggested that there were many unlisted ESSPF in the province. The inclusion criterion for the present study was pig farms with ≤ 50 sows, located within the five districts of Limpopo that have been active in pig production for at least one year (Mokoele *et al*. 2014). A participatory research model approach was used to collect information from the farmers (Raman, Sanghi & Chambers 1989).

Of the 185 ESSPFs sent questionnaires, a total of 164 (88.65%) participated fully and filled in the questionnaires completely. All the data were entered into Microsoft Excel^®^ and checked for consistency, correctness and validity. A preliminary map was drawn to check that all the places surveyed fell within the correct locations within Limpopo, based on the data obtained.

Data were formatted to meet the need for cartography software in ESRI's ArcGIS/ArcView, the R and the NodeXL software and exported appropriately. Extracts of the reports on the ASF outbreaks to date in South Africa were integrated into the final social network maps in order to predict the risk of ASF and advise on intervention strategies.

### Data analyses

#### Cartography, point mapping and one-way linkages

**FIGURE 1:**
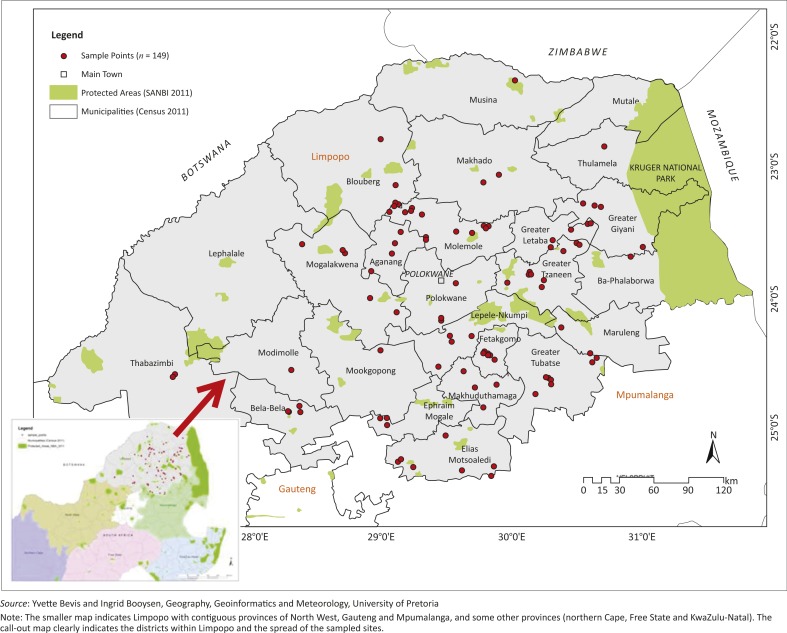
Map of surveyed Limpopo province and locations for emerging small-scale pig farmers in Limpopo, 2012.

**FIGURE 2:**
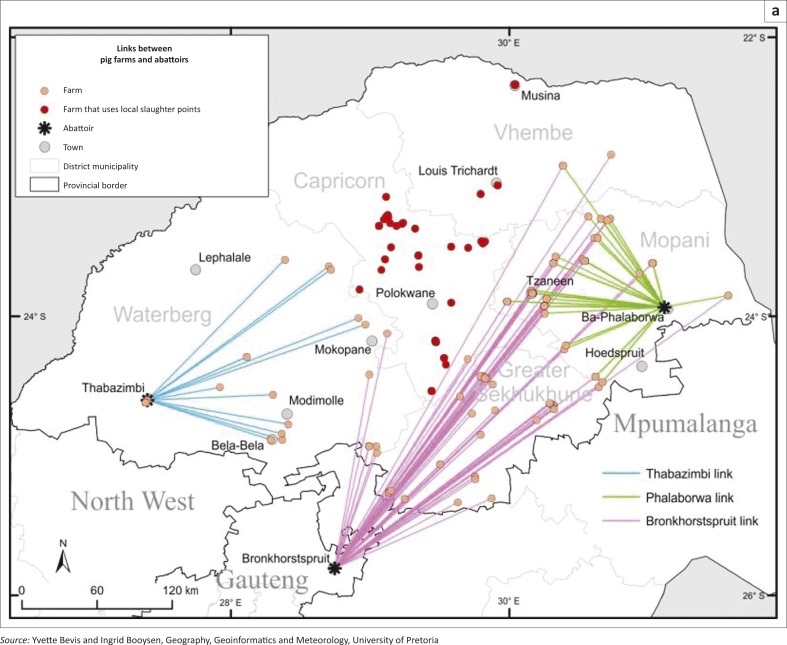

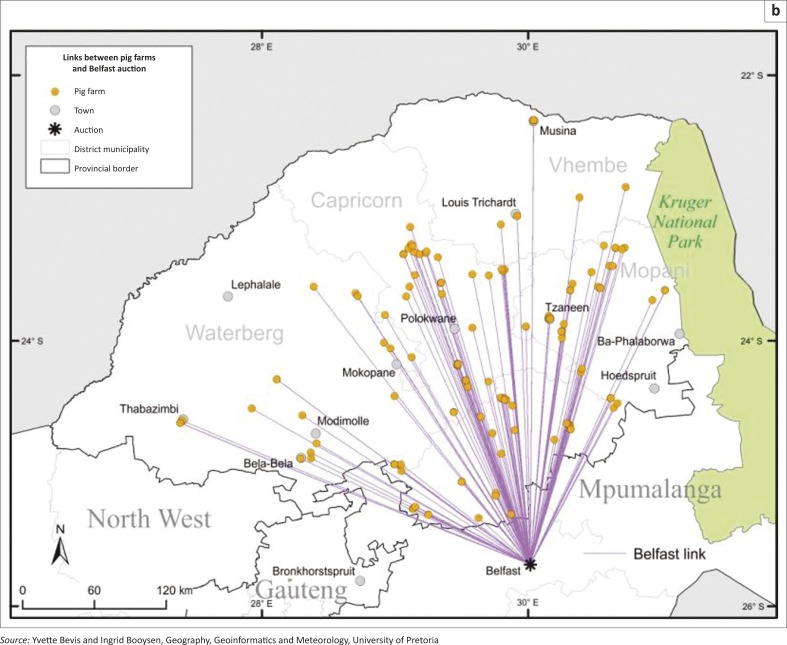
(a) Unidirectional links between ESSPF and destinations of final products. (b) Unidirectional links between ESSPF and preferred auction points.

**FIGURE 3:**
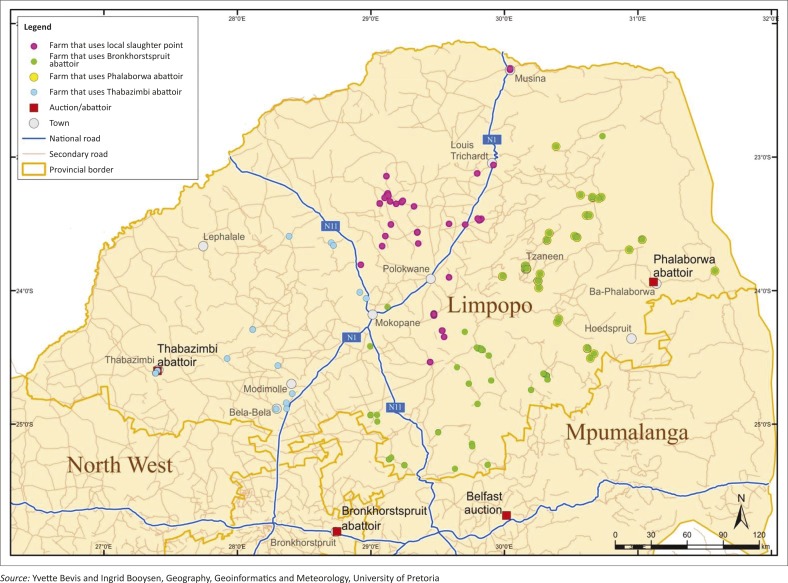
Distribution of locations of surveyed farms based on preferred slaughter location/auction markets and the national and secondary road networks.

ESRI's ArcGIS 10.1 software was used to add into the GIS all the specified geographic co-ordinates of the ESSPF farmers as XY co-ordinate data. The resulting event layer was then displayed as a point symbol (red dot) portraying the locations of the small-scale pig farmers on an administrative map of Limpopo (Fig. 1, Census 2011). Additional fields were added to the initial attribute table of the feature to enable the selection of farms by their designated abattoir. Thereafter the ‘*XY to Line*’ feature tool was used to construct geodetic lines or linkages (Fig. 2a and 2b) representing the shortest distance between the farm and the destination point (the abattoir or auction point). Pig farmers who used local slaughtering points within their localities were represented by symbols (red dots) on these two maps. In addition, layers of the primary and secondary road networks within Limpopo and contiguous provinces were made on the map previously generated (Fig. 3).

#### Social network analyses

**Unidirectional networks:** Firstly, filtered data were imported from the open Microsoft Excel^®^ workbook into the NodeXL environment and manipulated appropriately for analyses based on the software manufacturer's instructions (NodeXL version 1.0.1.326, Connected Action). Unidirectional graphs with 28 vertices were produced. The graph's vertices were grouped by cluster using the Clauset-Newman-Moore cluster algorithm and laid out using the Harel-Koren Fast Multiscale layout algorithm. The edge colours, widths and opacity were based on edge weight values. The vertex sizes were based on betweenness centrality (BC) values. For emphasis, a graph or network is a set of vertices and edges connected together [G = (V, E)], edge [E] is an association linking two vertices, and vertices [V] are points or locations joined by edges (Jiang *et al*. 2009).

**Multidirectional graphs:** In the second instance, data were exported into the R software environment and mapped for in-going (in-degrees) and out-going (out-degrees) contacts using the modified method of Nøremark and Widgren (2015). Multidirectional graphs were produced separately for in-degree and out-degree links. In addition, the k-cores (strength of contribution of each vertex in the total network) were produced based on the number of links that influence each vertex.

## Results

### Farm characteristics and trade relationship

A total of 164 ESSPF were categorised based on the number of sow units (SU) as follows: 1–10 SU (*n* = 124; median = 4, CI_95%_ = 4–5); 11–20 SU (*n* = 17; median = 15, CI_95%_ = 13–16); 21–30 SU (*n* = 7; median = 26, CI_95%_ = 23–29); 31–40 SU (*n* = 2; median = 38, CI_95%_ = 6–69); 41–50 SU (*n* = 1; median = not applicable, CI_95%_ = not applicable). An abattoir was the preferred slaughter point for the majority of respondents (105/164; 64%) and the abattoir of choice for the ESSPF from Limpopo was Bronkhorstspruit in Gauteng (55.9%) and Phalaborwa (26.2%) and Thabazimbi (10.4%), both located in Limpopo. A total of 97.6% of the surveyed ESSPF preferably chose the Belfast auction in Mpumalanga to source their pigs or sell whole animals (Fig. 2a and 2b). Importantly, some ESSPFs (34.1%), especially those from Capricorn district prefer to slaughter pigs within the communities or at pension points (Fig. 2a and 2b). Up to 306 pigs were marketed monthly (mean/month = 283) by the surveyed farmers and a total of 3396 pigs were sold in the year 2011 preceding the survey.

The ESSPFs were randomly dispersed throughout Limpopo, with a tendency for greater farm concentrations around Capricorn, Mopani and Greater Sekhukhune districts (Fig. 1). However, no specific pattern exists for the farm distributions or choice of slaughter or sale facility and for the distances from the closest national or secondary road(s) to the farms (Table 2; Fig. 3).

**TABLE 2:** Preferred point of sale for pigs and pig products and the distances to the nearest national or secondary roads.

Preferred sale point	Mean distance to secondary road (km)	Quartile (km)		Range		Mean distance to national road (km)	Quartile (km)		Range	
				Lower (25th)	Upper (75th)		Minimum	Maximum		Lower (25th)	Upper (75th)	Minimum	Maximum
Pension points and local slaughter (*n*= 55)	1.624 ± 1.859	0.345	2.080	0.046	2.127	29.727 ± 18.260	10.896	42.394	0.193	65.702
Bronkhorstspruit (*n*= 88)	3.092 ± 3.697	0.481	4.120	0.035	13.962	74.386 ± 38.686	49.919	98.638	2.676	77.242
Phalaborwa (*n*= 43)	4.728 ± 4.484	0.358	9.007	0.075	13.962	92.253 ± 30.956	69.280	112.454	49.919	201.289
Thabazimbi (*n*= 17)	1.434 ± 2.148	0.155	1.450	0.005	7.713	45.895 ± 46.169	5.540	67.577	1.211	116.209
Belfast auction (*n*= 161)	2.902 ± 3.565	0.358	4.113	0.005	13.962	63.833 ± 40.922	27.051	91.708	0.193	201.289

*Source*: Yvette Bevis and Ingrid Booysen, Geography, Geoinformatics and Meteorology, University of Pretoria

Note: The smaller map indicates Limpopo with contiguous provinces of North West, Gauteng and Mpumalanga, and some other provinces (northern Cape, Free State and KwaZulu-Natal). The call-out map clearly indicates the districts within Limpopo and the spread of the sampled sites.

The average distances between each of the slaughter or sale locations and the nearest national or secondary road networks are shown in Table 2. In addition, the distance covered to travel with the animals to the Belfast auction may be as short as 50 km up to a distance of about 400 km. The distances travelled to get to local abattoirs or slaughter points were within 5 km for local slaughter points, 150 km for Phalaborwa abattoir, 200 km for Thabazimbi abattoir and up to 400 km for Bronkhorstspruit abattoir (Fig. 2a and 2b; Mokoele *et al*. 2014). The preferred means of transportation includes own vehicle (34.5%), hired vehicle (5.4%), shared vehicle (1.4%) and other means (58.8%, *n* = 148).

### Social network analyses

**FIGURE 4:**
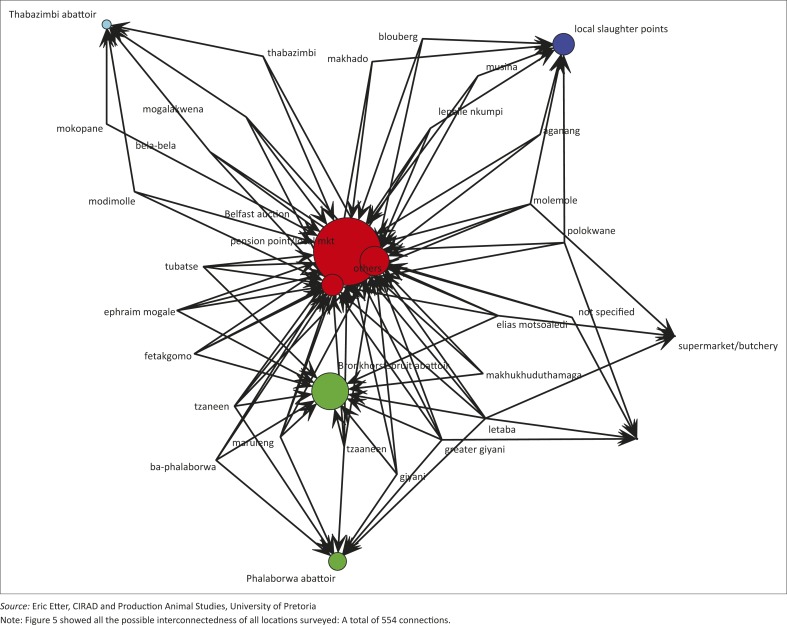
Network map of connected locations for emerging small-scale pig farmers in Limpopo.

Using the in-going (in-degree) and out-going (out-degree) movement to and from the vertices, the most important nodes for disease spread are locations (Phalaborwa, Tzaneen, Belfast auction, Bronkhorstspruit abattoir, Elias Motsoaledi, Molemole), pension points or local markets, Tubatse, local slaughter points and Polokwane in that order (Fig. 2a and 2b, 4–7). Phalaborwa also has the highest degree of centrality whereas the top ten vertices ranked by BC were the local slaughter points, Phalaborwa, Thabazimbi, Bronkhorstspruit, Molemole, Ephraim Mogale, Letaba, Tzaneen, Makhado and Blouberg (see Fig. 4 and Online Appendix 2).

Overall, the unidirectional graph has 28 vertices and 2 unique edges with a total of 161 edges. There were 4 connected components with an average geodesic distance (shortest path between points) of 1.48. The graph density (a function of the edges and vertices that make up a network) had a value of 0.06 and an average clustering coefficient (a measure of the degree to which nodes cluster together within the network) of 0.000 (Online Appendix 2).

## Discussion

This study revealed that informal pig movements and trade networks in Limpopo exist. While we did not investigate the specific role of each farmer and the middlemen, we confirmed that trade-associated short and long-distance movements of pigs and pig products exist within the province (5 km – 400 km). Monthly, up to 306 pigs may be transported individually or collectively in shared facilities over a short or long distance to major abattoirs or auction points and these transports may facilitate long-distance risk of disease dispersal. In a case of a rapidly spreading disease outbreak, trade by these farmers could facilitate the rapid spread of disease across broad geographic areas as was the case of the Lephalale-Sundra-Rietpoort (Limpopo-Mpumalanga-Gauteng) African swine fever outbreak of 2011–2012. It will be necessary to target the ESSPFs and associated downstream sector traders in this sector for animal health and biosecurity training. In addition, animal health intervention and prevention strategies should target these ESSPF (McCarron *et al*. 2015). Previous reports have confirmed that the majority of the past outbreaks of ASF have involved ESSPF from Limpopo (DAFF 2014).

**FIGURE 5:**
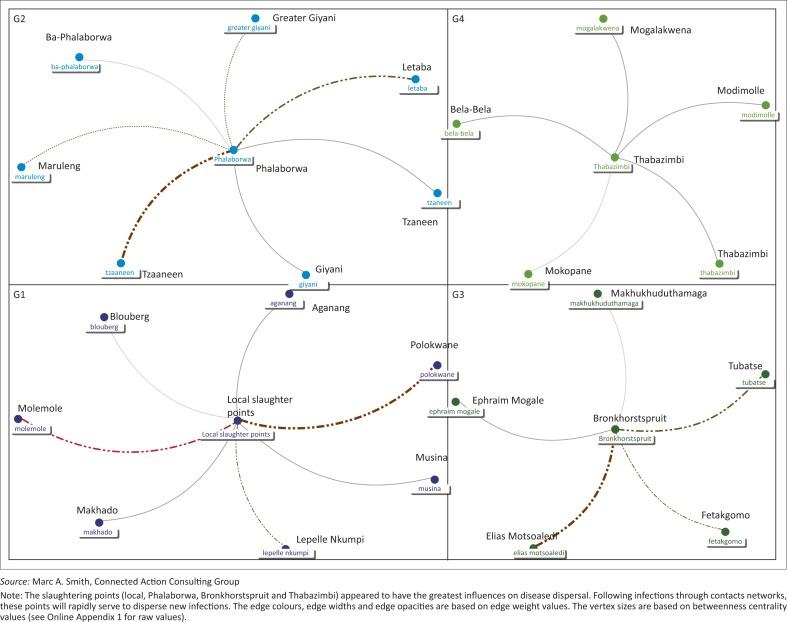
Multidirectional network map of in-degree connectedness of locations of emerging small-scale pig farmers in Limpopo, 2012.

Several important nodes (towns and cities) with high levels of interactions and the potential to contribute to or rapidly spread pig pathogens inadvertently were identified. These nodes acted as dispersal and sometimes collection points. Phalaborwa, Thabazimbi, Bronkhorstspruit and Belfast are among such important points and there is a need to create surveillance points and inspection nodes around these locations. The first two locations are within Limpopo but the last two are located in the adjacent Gauteng and Mpumalanga, where higher market values are the main drivers for the ESSPFs travelling these distances. It will be important to improve pig marketing locally and add value to pork products in Limpopo to discourage long-distance travel, because such trips can pose a risk of spread of infection outside the control area. Although a movement control policy is implemented in South Africa, which includes a prohibition of the movement of pigs out of the ASF control area without a veterinary permit only issued under specified conditions, emergency situations and economic imperatives often challenge such policies. There is therefore a need for enhanced vigilance on the part of the authorities, including active surveillance and participatory epidemiology among the ESSPFs.

In addition, there is a need to motivate ESSPF to apply good farming practice with improved biosecurity, which will be rewarded by access to a scaled-up pricing system for pork certified by local veterinarians through a government support system (Fasina *et al*. 2010, Fasina *et al*. 2012b; Logar 2014).

Since local slaughter and pension point sales were also revealed as important trade nodes, these activities may conceal the existence of subclinical infections and prevent early detection of rapidly spreading transboundary animal diseases. Limpopo is of major interest because the great majority of ASF outbreaks over decades have occurred or originated there. It is an important disease node not only for ASF but potentially for other pig diseases.

While the movement of pigs between farms was not evaluated in this study, we are aware that such movements exist (Mokoele *et al*. 2014). Social networks associated with pig farms are important factors in the transmission of infectious diseases (Leslie *et al*. 2015). Our model has identified local slaughter points and other abattoirs as potentially having the greatest influence on disease dispersal. Strict anticipatory planning should be implemented in these locations and controlled slaughter should be encouraged (Kao *et al*. 2007; Lindström *et al*. 2010).

Our evaluation and analyses are based on certain assumptions. First, we assumed that the records are complete and the data used in the evaluations are accurate; it is important for recording officials to pay particular attention to data entry in the future, as critical analysis based on available data may be warranted. We have made every effort to verify the data and confirm certain facts where there are doubts; secondly, we did not discriminate between respondents, partial respondents and non-respondents as we eliminated all partially filled-in questionnaires. This resulted in differing denominators and may sometimes hide some important answers. Some degree of recall bias may have occurred with regard to certain questions that made reference to the past years. Moreover, since only the surveyed farmers were included in the networks, it is possible that some other smallholder pig farmers exist whose inclusion may significantly influence and change the strengths and directions of the networks. Thirdly, the social network analytic method used in this study has certain limitations including the following:

- An assumption that all disease algorithms often exhibit ‘small world’ properties in a situation of close interconnectedness in temporal and spatial locality (Jiang *et al*. 2009); in the real world this may not always be true.
- An assumption that an analysis kernel of BC can resolve all complexities of interconnectedness by measuring the shortest distances passing through vertices (Freeman 1977).
- The networks can sometimes identify false positive edges and false negatives especially where background noises are high in the dataset (Ufimtsev & Bhowmick 2013). False positives may arise when two vertices have low to medium BC scores, which can combine to produce one with a high BC score. We have reduced this by creating four different networks for each identified node.
- Finally, the threshold in a given group testing may be affected by the false positive BC values. We have corrected for this influence in the course of the modelling.

**FIGURE 6:**
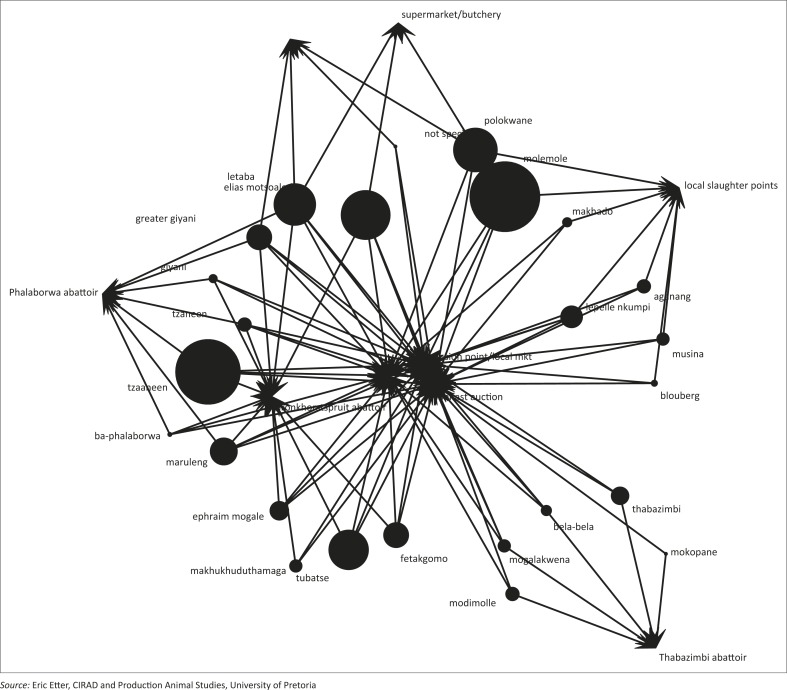
Multidirectional network map of out-degree connectedness of locations of emerging small-scale pig farmers in Limpopo, 2012.

In conclusion, we have produced evidence that geographic factors and trade-movement networks played roles in ASF dynamics and dispersal in South Africa and that managing these in Limpopo province is critical for pig disease control. The degree of interconnectedness of the ESSPF in the province may facilitate disease spread by people, trade, animal movements and also possible sharing of transport and farm equipment as has been describe elsewhere (Kao *et al*. 2007; Leslie *et al*. 2015). While we have made effort to integrate the dynamics of recent and historical outbreaks of ASF in South Africa geo-temporally, it will be worthwhile to re-evaluate the specifics of the Lephalale-Sundra-Rietpoort outbreak of 2011–2012 and determine quantitatively the potential implications of ‘*along-the-road networks*’, the effect of delayed response due to travel time to auction time and treatment using antibiotics. These may reveal important hidden evidence of disease dynamics in the pig industry in South Africa

**FIGURE 7:**
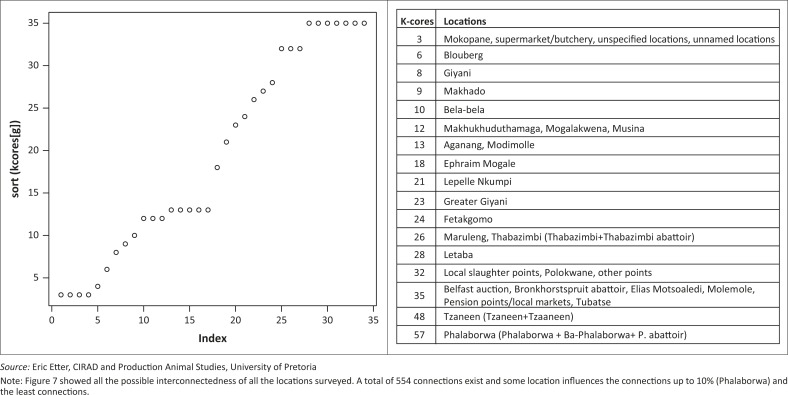
Structural cohesion (k-core) of network map of connected locations of emerging small-scale pig farmers in Limpopo, 2012.
